# Association between medication adherence and illness perceptions in atrial fibrillation patients treated with direct oral anticoagulants: An observational cross-sectional pilot study

**DOI:** 10.1371/journal.pone.0204814

**Published:** 2018-09-28

**Authors:** Motoyasu Miyazaki, Akio Nakashima, Yoshihiko Nakamura, Yuya Sakamoto, Koichi Matsuo, Miwa Goto, Masanobu Uchiyama, Keisuke Okamura, Ryoko Mitsutake, Hidenori Urata, Hidetoshi Kamimura, Osamu Imakyure

**Affiliations:** 1 Department of Pharmacy, Fukuoka University Chikushi Hospital, Fukuoka, Japan; 2 Department of Emergency and Critical Care Medicine, Faculty of Medicine, Fukuoka University, Fukuoka, Japan; 3 Department of Cardiovascular Diseases, Fukuoka University Chikushi Hospital, Fukuoka, Japan; 4 Department of Pharmacy, Fukuoka University Hospital, Fukuoka, Japan; Inselspital Universitatsspital Bern, SWITZERLAND

## Abstract

**Objective:**

The aim of this study was to examine the association between medication adherence and illness perceptions, and to explore the factors associated with poor medication adherence in atrial fibrillation (AF) patients receiving direct oral anticoagulants (DOACs) in a real-world clinical setting.

**Methods:**

An observational cross-sectional pilot study was conducted at a single Japanese university hospital. One hundred and twenty-nine patients who were diagnosed with AF and who were taking DOACs were recruited from outpatients between January 4^th^ and April 25^th^, 2017. We evaluated medication adherence to DOACs using the Morisky Medication Adherence Scale-8 (MMAS-8) and illness perceptions using the Brief Illness Perception Questionnaire (BIPQ). The patients’ characteristics and clinical data were collected from electronic medical records.

**Results:**

Ninety-nine (76.7%) patients (male, n = 74; mean age, 71.4±9.8 years) participated in this study. According to the MMAS-8, 21 (21.2%) of the patients were classified into the poor adherence group (MMAS-8 score of <6), and 78 (78.8%) were classified into the good adherence group (MMAS-8 score of 6–8). A multivariate logistic regression analysis revealed that age (per year, odds ratio [OR] 0.912, 95% confidence interval [CI] 0.853–0.965, p = 0.001), a history of warfarin use (OR 0.181, 95% CI 0.033–0.764, p = 0.019), duration of DOAC exposure (per 100 days, OR 1.245, 95% CI 1.084–1.460, p = 0.001), and the BIPQ emotional response score (per 1 point, OR 1.235, 95% CI 1.015–1.527, p = 0.035) were significantly associated with poor medication adherence in AF patients receiving DOACs.

**Conclusion:**

Poor medication adherence to DOACs was strongly associated with a stronger emotional response (i.e. stronger feelings of anger, anxiety, and depression), as well as younger age, the absence of a history of warfarin treatment, and longer DOAC exposure. Further evaluation of the factors associated with medication adherence in AF patients and the development and execution of strategies for improving poor adherence are warranted in the real-world clinical setting.

## Introduction

Non-valvular atrial fibrillation (AF) is known as one of the most important risk factors for stroke. Data from the Japanese Circulation Society show that in 2005, approximately 0.7 million people suffered from AF in Japan; by 2050, the number is predicted to increase to 1 million (1.09% of the Japanese population) [1]. Until recently, warfarin was the only agent used to prevent stroke in AF; however, direct oral anticoagulants (DOACs), including dabigatran, rivaroxaban, apixaban, and edoxaban, have been approved for anticoagulation therapy in Japan since 2011. A meta-analysis of clinical trials revealed that DOAC therapy reduced stroke or systemic embolic events by 19% and all-cause mortality by 10% [2]. Unlike warfarin, DOACs have many pharmacological advantages, including a rapid onset/offset of action and a short half-life, predictable pharmacokinetics and pharmacodynamics without the need to constantly monitor the prothrombin time–international normalized ratio (PT-INR), a good safety profile, and fewer drug and food interactions [3]; on the other hand, there have some disadvantages, including high cost and potentially lower treatment motivation due to no PT-INR monitoring as therapeutic index. Therefore, physicians should decide which to prescribe DOACs or warfarin for oral anticoagulant (OAC) therapy depending on the patient’s clinical and psychological characteristics.

Non-adherence to medication is one of the most important drug-related issues. Adherence to OACs in AF patients is also essential from the viewpoint of clinical efficacy and safety [4]. Shore et al. reported that lower medication adherence to dabigatran was associated with an increased risk for combined all-cause mortality and stroke [5]. Although medication adherence to OACs has been reported in some studies, it is currently controversial because of heterogeneity in the settings and the methods employed to evaluate medication adherence, including the proportion of days covered (PDC), the Morisky Medication Adherence Scale, and other methods [6–10]. Illness perceptions are modifiable factors and have been reported to be a major factor that influences medication adherence in patients with chronic disease [11]. Broadbent et al. developed the Brief Illness Perception Questionnaire (BIPQ) to assess appropriate psychometric properties [12]. Until recently, some reports have indicated that medication adherence was associated with illness perceptions in some diseases [12–16], but not in AF. A previous study by Clarkesmith et al. demonstrated that warfarin-treated AF patients who received some educational intervention for improving their medication adherence achieved a significantly better improvement of time within the therapeutic range in comparison to patients treated with usual care [17]. Educational intervention for patients who have some factors associated with poor medication adherence may improve the clinical outcome and the safety of their treatment. However, no studies have examined the patients’ characteristics, including illness perceptions, and the clinical factors associated with poor medication adherence, especially in AF patients receiving DOACs.

The aim of this study was to examine the association between medication adherence and illness perceptions, and to explore the factors associated with poor medication adherence in AF patients receiving DOACs in a real-world clinical setting.

## Materials and methods

### Study design and population

A questionnaire-based, observational cross-sectional pilot study was conducted at a single Japanese university hospital. Patients who were diagnosed with AF and who were taking DOACs (dabigatran, rivaroxaban, apixaban, or edoxaban) were recruited from outpatients who attended the Department of Cardiovascular Diseases between January 4^th^ and April 25^th^, 2017. The outpatients were seen at least once within a four-month period. Since the baseline clinical characteristics of patients who were taking warfarin were assumed to be different from those of patients receiving DOACs, patients who were prescribed warfarin were excluded from this study. Patients were excluded if they were <20 years of age, if they did not self-administer their medications, or if they did not agree to participate in the study. This study was approved by the Fukuoka University-Medical Ethics Review Board (R15-026). All participants provided their written informed consent before enrollment.

### Assessment of medication adherence

Medication adherence was assessed by the Morisky Medication Adherence Scale-8 (MMAS-8), which was developed by Morisky et al—a widely-used self-report questionnaire [18]. The first seven items are Yes/No responses and the last item is a 5-point Likert response. The MMAS-8 has proven quite useful in clinical practice for its capture of the fundamental reasons of underuse, such as forgetfulness, considering the circumstances related to adherence behavior of patients. We have obtained written permission from Donald E. Morisky to use the MMAS-8. According to the MMAS-8, adherence was categorized as high adherence (a score of 8), medium adherence (a score of 6 to <8) and low adherence (a score of <6). The MMAS-8 has been reliable (alpha reliability = 0.83) for the assessment of medication adherence in outpatients with hypertension, and using a cut-point of 6, it was significantly associated with blood pressure control [18], showing that the MMAS-8 was enough to validly screen a patient’s medication adherence. To explore the factors associated with poor medication adherence, the patients were divided into two groups: the poor adherence group (a score of < 6) and the good adherence group (a score of 6–8) [18–20]. We also investigated the PDC in patients who has been prescribed DOACs for at least continuous six months without admission before enrollment. PDC was defined as the proportion of days in which medication was available to the patients in a six-month period.

### Assessment of illness perceptions

Illness perceptions were evaluated by the BIPQ [12]. This 9-item questionnaire was designed to assess a patient’s cognitive and emotional representations of illness on an 11-point (score 0–10) Likert scale. The questionnaire includes 8 dimensions (items 1 to 8): consequences, timeline, personal control, treatment control, identity, concerns, understanding, and emotional response. Item 9 of the BIPQ is an open question that explores causal representation, which was not considered in this study. The overall score was calculated as the sum of the reversed scores of items 3, 4, and 7 and the scores of items 1, 2, 5, 6, and 8. Thus, a higher BIPQ overall score reflects that the patient views the illness as more threatening.

### Data collection

The patients’ characteristics, clinical information, and prescription information were collected from electronic medical records. The characteristics included gender, age, height, weight, and body mass index (BMI). Current smoking and drinking status, admission within 1 year, history of adverse effects from any drugs, and whether the patient lived alone were collected via interviews with the patients, as well as medical records. Clinical information included comorbidities (heart failure, hypertension, angina pectoris, chronic obstructive pulmonary disease/bronchial asthma, diabetes mellitus (DM), chronic kidney disease, insomnia, cancer, history of myocardial infarction and cerebral infarction), implantation of medical devices (i.e. an implantable cardiac defibrillator or pace maker), CHADS_2_ score at enrollment. The patients’ prescription information included the following information: one dose package (ODP), prescription by medical specialists other than physicians of the Department of Cardiovascular Diseases, number of medicines, polypharmacy (number of oral medications ≥6), number of administrations, combination use of antiplatelet, prescription of non-oral medication, history of warfarin use, and duration of DOAC exposure.

### Statistical analyses

The internal consistency of the test scores of the MMAS and BIPQ were assessed using Cronbach’s alpha coefficients. An acceptable Cronbach’s alpha value is ≥0.7 [21]. Concurrent validity was assessed through the association between the MMAS-8 score and the PDC by Spearman’s correlation coefficient, and the cut-point MMAS-8 score of 6 was validated by the significant difference of the mean PDC value between the poor adherence group and the good adherence group. Binary variables were expressed as the proportion and normally distributed continuous variables were expressed as the means and standard deviation. To determine the factors associated with poor adherence, including the patients’ characteristics, clinical factors, and illness perceptions, univariate analysis was conducted using the chi-squared test or Fisher’s exact test (as appropriate) for proportions and the two-tailed t-test for means. Factors that were associated (p<0.1) with poor adherence in the univariate analysis were included in the multivariate logistic regression analysis with a stepwise procedure to identify risk factors that were independently associated with poor adherence. The BIPQ overall score was not included in the multivariate analysis, as we investigated the influence of each illness perception item on medication adherence. The JMP software program (version 10, SAS Institute Inc., Cary, NC, USA) was used for all the statistical analyses. P-values of <0.05 were considered to indicate statistical significance.

## Results

### Patients’ characteristics and clinical data

**Fig 1:**
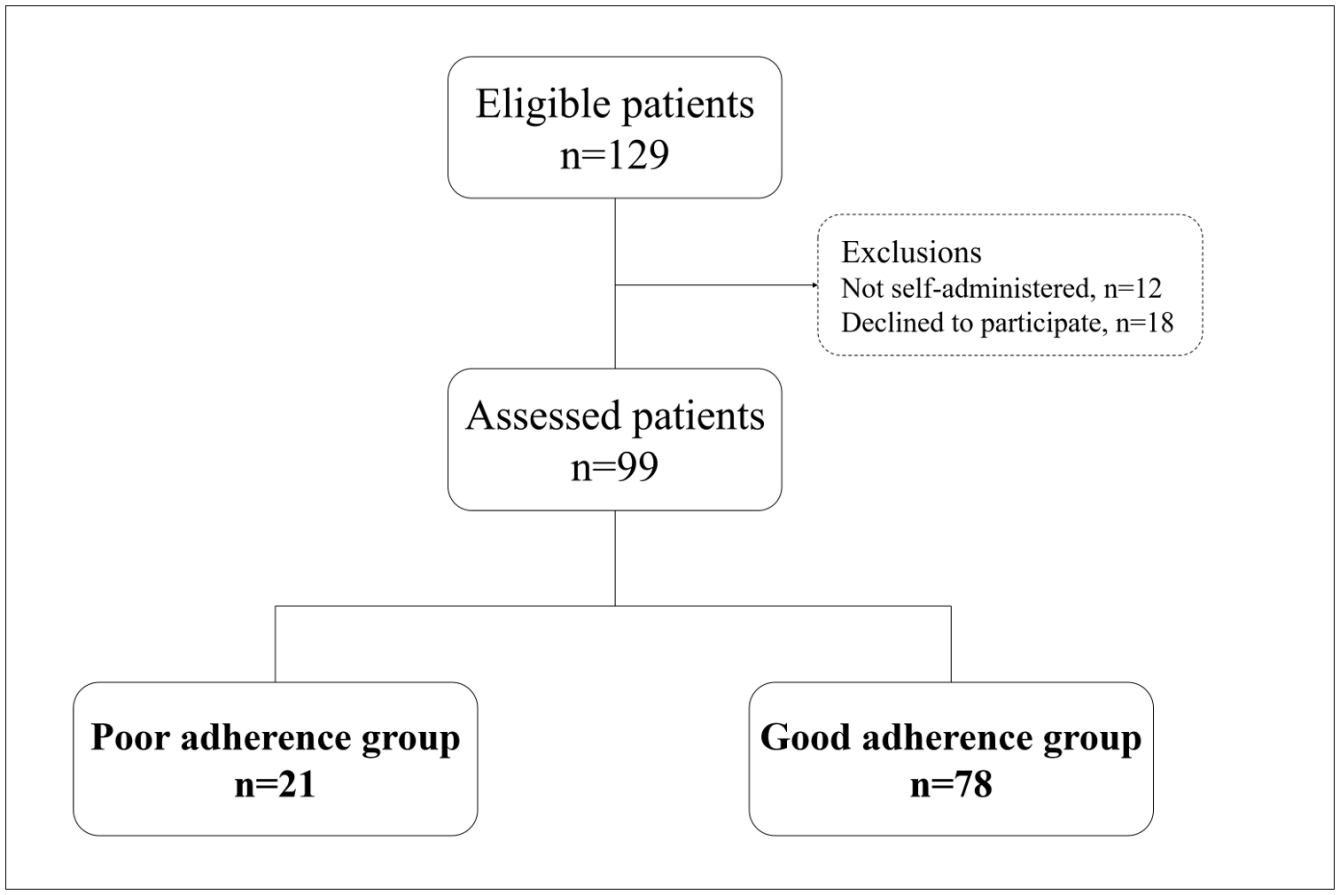
A flow diagram of this study. We excluded 18 patients who declined to participate and 12 patients who did not self-administer their medicines. The 99 patients were divided into the poor adherence and good adherence groups according to their MMAS-8 scores.

Between January 4^th^ and April 25^th^, 2017, 129 patients were eligible to participate in this study. Eighteen (14.0%) patients declined to participate in this study and 12 (9.3%) patients did not self-administer their medications (Fig 1). As a result, 99 (76.7%) patients completed the MMAS-8 and BIPQ (S1 and S2 Tables).

The patient characteristics, clinical information, and prescription information are listed in Table 1. Seventy-four (74.7%) of the patients were male; the mean age was 71.4±9.8 years; 82 (82.8%) patients were ≥65 years of age; and 17 (17.4%) lived alone. Fifteen percent and forty-three percent of the patients were current smokers and drinkers, respectively. The most frequently prescribed DOAC was rivaroxaban (n = 38, 38.4%), followed by apixaban (n = 30, 30.3%), dabigatran (n = 19, 19.2%), and edoxaban (n = 12, 12.1%). The mean duration of DOAC exposure was 625.0±461.2 days. Approximately 70% of the patients had hypertension, and 40% had heart failure as comorbidities. The mean CHADS_2_ score was 1.9±1.3. More than 60% of the patients were prescribed ≥6 drugs for several conditions, which fulfilled the definition of polypharmacy. Thirty-seven (37.4%) of 99 patients had used warfarin before using DOACs.

**Table 1:** The patient characteristics, clinical information, and prescription information.

Variables		n = 99	%
**Patient characteristics**			
Gender	Male	74	74.7
Mean (SD) age in years		71.4	9.8
	<65 years of age	17	17.2
	65–74 years of age	43	43.4
	75< years of age	39	39.4
Mean (SD) BMI^a^		24.0	4.2
Smoking^b^		13	15.1
Drinking^c^		37	43.0
Admission within 1 year		35	35.4
History of adverse effects		17	17.2
Single-living^d^		16	17.4
**Clinical information**			
Mean (SD) CHADS_2_ score		1.9	1.3
Comorbidity	Heart failure	39	39.4
	Hypertension	70	70.7
	Angina pectoris	32	32.3
	COPD/BA	6	6.1
	DM	28	28.3
	CKD	11	11.0
	Insomnia	15	15.2
	Cancer	17	17.2
	History of MI	9	9.1
	History of CI	9	9.1
ICD/PM		11	11.1
**Prescription information**			
ODP		27	27.3
DOAC	Dabigatran	19	19.2
	Rivaroxaban	38	38.4
	Apixaban	30	30.3
	Edoxaban	12	12.1
Dosing	Once-daily dosing	53	53.5
	Twice-daily dosing	46	46.5
Prescription by other medical specialists		23	23.2
Mean (SD) number of medicines		6.7	3.4
	Polypharmacy	61	61.6
Mean (SD) number of administrations		2.3	1.0
Combination use of antiplatelet		19	19.2
Prescription of non-oral medication		26	26.3
History of warfarin use		37	37.4
Mean (SD) duration (days) of DOAC exposure		625.0	461.2

Number assessed,

^a^: n = 98,

^b^: n = 86,

^c^: n = 86,

^d^: n = 92.

BA, bronchial asthma; BMI, body mass index; CI, cerebral infarction; CKD, chronic kidney disease; COPD, chronic obstructive pulmonary disease; DM, diabetes mellitus; DOAC, direct oral anticoagulant; ICD, implantable cardiac defibrillator; MI, myocardial infarction; ODP, one dose package; PM, pace maker; SD, standard deviation.

### The assessment of medication adherence and illness perceptions

The mean MMAS-8 and BIPQ overall score were 6.8 and 32.3, respectively. The Cronbach’s alpha coefficients of the MMAS-8 and BIPQ were 0.705 and 0.707, respectively (S3 and S4 Tables). According to the MMAS-8, 21 (21.2%) of the patients were classified into the poor adherence group, and 78 (78.8%) were classified into the good adherence group. Medication adherence could be assessed by the PDC in 61 patients. The mean PDC and MMAS-8 of these patients were 98.0% and 6.7, respectively. Spearman’s rank correlation coefficient was 0.380 (p = 0.003). The mean PDC of the patients in the poor adherence group was significantly lower than that in the good adherence group (92.8% vs. 98.9%, p<0.001).

### The association between medication adherence and illness perception

Table 2 shows the illness perception of the patients in the poor and good adherence groups. The poor adherence group showed significantly lower scores for personal control (5.5±2.0 vs. 6.9±2.4, p = 0.022) and treatment control (7.1±1.8 vs. 8.3±2.1, p = 0.020), and tended to show a higher score for emotional response (4.0±2.7 vs. 2.8±2.8, p = 0.086) in comparison to good adherence group. The mean BIPQ overall score in the poor adherence group was tended to be higher than that in the good adherence group (36.9±10.4 vs. 31.0±12.4, p = 0.051).

**Table 2:** The association between medication adherence and illness perception.

Items	Poor adherence, n = 21		Good adherence, n = 78		P value
	Mean	SD	Mean	SD	
Consequences	4.6	2.7	3.8	3.3	0.312
Timeline	7.9	2.0	8.1	2.8	0.764
Personal control	5.5	2.0	6.9	2.4	0.022
Treatment control	7.1	1.8	8.3	2.1	0.020
Identity	2.9	2.4	3.1	2.8	0.809
Concerns	6.9	2.0	5.8	3.3	0.177
Understanding	6.7	1.7	7.4	2.4	0.247
Emotional response	4.0	2.7	2.8	2.8	0.086
BIPQ overall score	36.9	10.4	31.0	12.4	0.051

BIPQ, Brief Illness Perception Questionnaire; SD, standard deviation.

### The univariate analysis of clinical factors associated with poor medication adherence

Table 3 shows the patient characteristics and clinical factors that were associated with poor medication adherence in the univariate analysis. The patients in the poor adherence group were significantly younger (65.0±11.8 vs. 73.2±8.5, p<0.001), fewer patients tended to have a history of warfarin use (19.1% vs. 42.3%, p = 0.074) and the duration of DOAC exposure tended to be longer (782.9±389.7 days vs. 582.5±471.9 days, p = 0.077) in comparison to the good adherence group; however, these results did not reach statistical significance. The CHADS_2_ score, comorbidity, prescription information (i.e. ODP, polypharmacy, and number of administrations) were not associated with medication adherence.

**Table 3:** The clinical factors associated with poor medication adherence in the univariate analysis.

Variables		Poor adherence, n = 21		Good adherence, n = 78		P value
		n	%	n	%	
**Patient characteristics**						
Male gender		18	85.7	56	71.8	0.262
Mean (SD) age in years		65.0	11.8	73.2	8.5	<0.001
Mean (SD) BMI^a^		24.7	5.9	23.9	3.6	0.430
Smoking^b^		5	29.4	8	11.6	0.122
Drinking^c^		8	50.0	29	41.4	0.532
Admission within 1 year		6	28.6	29	37.2	0.464
History of adverse effect		3	14.3	14	18.0	>0.999
Single-living^d^		4	21.1	12	16.4	0.735
**Clinical information**						
Mean (SD) CHADS_2_ score		1.9	1.4	2.0	1.2	0.706
Comorbidity	Heart failure	9	42.9	30	38.5	0.714
	Hypertension	13	61.9	57	73.1	0.318
	Angina pectoris	4	19.1	28	35.9	0.192
	COPD/BA	0	0	6	7.7	0.337
	DM	7	33.3	21	26.9	0.563
	CKD	4	19.1	7	9.0	0.239
	Insomnia	4	19.1	11	14.1	0.732
	Cancer	2	9.5	15	19.2	0.514
	History of MI	2	9.5	7	9.0	>0.999
	History of CI	3	14.3	6	7.7	0.395
ICD/PM		2	9.5	9	11.5	>0.999
**Prescription information**						
ODP		5	23.8	22	28.2	0.387
Dosing	Once-daily dosing	10	47.6	43	55.1	0.540
	Twice-daily dosing	11	52.4	35	44.9	
Prescription by other medical specialists		3	14.3	20	25.6	0.788
Mean (SD) number of medicines		6.0	3.3	6.9	3.4	0.242
	Polypharmacy	11	52.4	50	64.1	0.327
Mean (SD) number of administrations		2.0	0.8	2.4	1.0	0.105
Prescription of non-oral medication		4	19.1	22	28.2	0.578
Combination use of antiplatelet		3	14.3	16	20.5	0.756
History of warfarin use		4	19.1	33	42.3	0.074
Mean (SD) duration (days) of DOAC exposure		782.9	389.7	582.5	471.9	0.077

Number of poor vs. good adherence,

^a^: 21 vs. 77;

^b^: 17 vs. 69;

^c^: 16 vs. 70;

^d^: 19 vs. 73.

BA, bronchial asthma; CI, cerebral infarction; CKD, chronic kidney disease; COPD, chronic obstructive pulmonary disease; DM, diabetes mellitus; DOAC, direct oral anticoagulant; ICD, implantable cardiac defibrillator; MI, myocardial infarction; ODP, one dose package; PM, pace maker; SD, standard deviation.

### A multivariate analysis of the factors associated with poor medication adherence

In the multivariate logistic regression analysis, age (per year, odds ratio [OR] 0.912, 95% confidence interval [CI] 0.853–0.965, p = 0.001), history of warfarin use (OR 0.181, 95% CI 0.033–0.764, p = 0.019), duration of DOAC exposure (per 100 days, OR 1.245, 95% CI 1.084–1.460, p = 0.001), and emotional response score (per 1 point, OR 1.235, 95% CI 1.015–1.527, p = 0.035) were significantly associated with poor medication adherence in AF patients treated with DOACs (Table 4).

**Table 4:** The factors associated with poor medication adherence in the multivariate analysis.

Predictor variables	OR	95% CI	P value
Age	0.912	0.853–0.965	0.001
History of warfarin use	0.181	0.033–0.764	0.019
Duration of DOAC exposure^a^	1.245	1.084–1.460	0.001
BIPQ emotional response score^b^	1.235	1.015–1.527	0.035

BIPQ, Brief Illness Perception Questionnaire; CI, confidence interval; DOAC, direct oral anticoagulant; OR, odds ratio.

^a^OR per 100 days increase.

^b^OR per 1 point increase.

## Discussion

In this cross-sectional pilot study, we found that younger age, a lack of previous warfarin treatment, a longer duration of DOAC exposure, and a stronger emotional response (i.e. feelings of anger, anxiety, and depression resulting from AF) were significantly associated with poor adherence in AF patients. To the best of our knowledge, this is the first study to demonstrate a relationship between adherence to DOACs and illness perceptions in AF patients.

In this study, the Cronbach’s alpha coefficient of the MMAS-8 was 0.705, which reflected acceptable reliability. Then, a significant correlation between the MMAS-8 scores and the PDC was found, and the MMAS-8 cut-point of 6 was validated based on the significant difference in the mean PDC value between poor adherence group and good adherence group. Our results show that the Japanese version of the MMAS-8 had an acceptable level of reliability and validity in AF patients receiving DOACs. However, the use of self-reported questionnaires may not reflect the true incidence because of the existence of recall bias among respondents. Further studies are needed to evaluate the relationship between the MMAS-8 and other methodologies involving direct (e.g. serum drug concentration) or indirect (e.g. pharmacy refill rates and pill counts) assessments of medication adherence. Broadbent et al. reported that the BIPQ showed good test–retest reliability in renal disease patients and concurrent validity with relevant measures in a variety of illnesses [12]. In our study, the Cronbach’s alpha coefficient of the BIPQ was 0.707, showing that the Japanese version of the BIPQ had an acceptable level of reliability in AF patients receiving DOACs; however, studies of its association with the outcome, disease severity, and quality of life were not performed in this study or in Japan. Further validation studies of BIPQ are therefore needed.

Some previous reports have described risk factors associated with poor adherence in relation to OAC therapy in AF patients. Non-adherence to warfarin appears to be more prevalent among younger patients [22, 23], which is in line with our results in AF patients treated with DOACs. In our study, an analysis stratified by age demonstrated that the prevalence of poor adherence among the patients in the adult group (<65 years of age) was higher than that among patients in the old group (>75 years of age) (38.1% vs. 19.1%, p = 0.004). Further studies that consider the lifestyle of patients would be needed. Interestingly, our results demonstrated that the percentage of patients with a history of warfarin use in the good medication adherence group was higher than that in the poor adherence group. A recent report by Manzoor et al. studying medication adherence in AF patients newly initiating a DOAC showed that the rates of PDC and persistence in anticoagulation-experienced patients were significantly higher in comparison to anticoagulation-naïve patients [24], this finding is in accordance with our results. One possible explanation as to why the patients with a history of warfarin use showed better adherence to OAC therapy could be that the medication management that is used when administrating warfarin is much stricter than used when administrating DOACs, from the viewpoints of routine monitoring of the PT-INR, food-drug interactions, and drug-drug interactions, leading to a high level of consciousness regarding the disease and therapy. In our sub-analysis to compare the BIPQ values of warfarin user and non-users, the mean timeline perception (whether an illness was acute, chronic, or cyclic duration) score of warfarin users was higher than that of warfarin non-users (8.9±2.0 vs. 7.5±2.8, p = 0.005); thus, patients with a history of warfarin use might have considered their illness to be chronic. Patients switching from warfarin to DOACs may have a longer perceived illness duration, which could heighten their adherence to OAC therapy. Similarly to medication adherence, persistence defined as the percentage of patients who continue therapy is a very important element for ensuring efficacy and safety in OAC therapy. In one study, which was focused on primary care patients, the probabilities of persistence after 180 and 360 days were 66.0% and 53.1% for rivaroxaban, and 60.3% and 47.3% for dabigatran, respectively [25]. This is supported by our results, which showed that among patients with poor adherence, the duration of DOAC exposure was longer in comparison to patients with good adherence. Broadbent et al. indicated that hospitalized myocardial infarction patients in whom a diagnosis had not been confirmed showed a higher emotional response, describing a high fear response to an unknown health threat [12]; these findings might be supported by our result, the association between poor adherence and higher scores for emotional response.

### Implications for clinical practice

The findings in this study can help tailor health care providers’ interventions in AF patients that may be at risk for poor adherence to DOACs. We identified four risk factors associated with poor adherence, which were either modifiable (i.e. perceived emotional response) or non-modifiable (i.e. younger age, the absence of a history of warfarin use, and a longer duration of OAC therapy). Why younger patients show a poorer adherence to medication than older patients are unclear; however, some younger patients forget to take their medicine on occasion due to irregular patterns of lifestyle, such as overworking companies’ employees. Regularly scheduled monitoring and contact with health care providers (e.g. telephone intervention) may improve their medication adherence. Patients without a history of warfarin use and with a longer duration of therapy showed decreased adherence and persistence [25]. The continuous evaluation of patients’ preference and educational intervention regarding the need for OACs, the risks and benefits associated with OAC therapy, and a medication regimen using the patient information card recommended by European Heart Rhythm Association can improve their knowledge, motivation, and skills for self-management [26]. On the other hand, modifiable factors, such as illness perceptions, have changed during treatment [17]. Psycho-educational intervention as well as intervention focused on the medical, physical, social, and economic implications may improve patients’ knowledge, treatment coherence, and medication brief, especially among patients with negative emotions resulting from chronic disease such as AF [27, 28]. An interdisciplinary team consisting of clinicians, pharmacists, nurses, or clinical psychotherapist should be responsible for the initial and continuous education in AF patients.

### Study limitations

This study is associated with several limitations. First, this was a pilot study conducted in a single university hospital, and the population was relatively small, which might have led to a selection bias in the process of gathering the study population. Participants may have been highly motivated regarding OAC therapy, as was seen in the high PDC and MMAS-8 scores. Second, non-psychological characteristics, such as income and working, were not considered. Third, we could not indicate the degree of adherence or illness perceptions in relation to clinical efficacy and safety in AF patients treated with DOACs.

### Conclusions

In a real-world clinical setting, poor medication adherence to DOACs was significantly associated with a stronger emotional response regarding AF, as well as younger age, the absence of a history of warfarin use, and a longer duration of DOAC use in AF patients. Interdisciplinary intervention by pharmacists, clinicians, nurses, or clinical psychotherapist for patients with factors associated with poor medication adherence may help improve their adherence and/or outcomes. The further evaluation of the factors associated with adherence in AF patients and the development and execution of strategies to improve poor adherence are warranted.

## Supporting information

S1 Table The results of MMAS-8. (XLSX)

S2 Table The results of BIPQ. (XLSX)

S3 Table The mean, standard deviation, and Cronbach’s alpha coefficients of MMAS-8. (XLSX)

S4 Table The mean, standard deviation, and Cronbach’s alpha coefficients of BIPQ. (XLSX)
